# The Story of the Insane from Year to Year

**Published:** 1897-07-10

**Authors:** 


### THE STORY OF THE INSANE FROM YEAR TO YEAR.

Tenth Series.

### EAST SUSSEX ASYLUM AT HAYWARD'S HEATH.

On the first day of January, 1896, this asylum contained 847 patients, and on the last day of the year the number had risen to 875. There were 198 first admissions, 39 readmissions, 76 discharged recovered, 42 discharged relieved, and 86 deaths. The recoveries stand at 33·1 per cent. on the admissions, and the deaths at 9·9 per cent. on the average number resident. Forty-five of the deaths were caused by cerebral and spinal diseases, 12 by consumption, and 2 by pneumonia. Of the year's admissions, 20 were driven insane by drink, 61 by hereditary influences, 6 by religion, and in 62 the cause was unknown. Although a few years only have elapsed since East Sussex dissolved partnership with West Sussex, more space in the old asylum is again called for; but it has not yet been decided how this call has to be met. In discussing the causes of insanity, Dr. Saunders says, "The part which hereditary predisposition plays in the causation of lunacy is recognised and accepted, but the extent to which it is manifested, even in one generation, is, of course, not generally known." And after referring to numerous striking cases coming under his own observation, he adds, "It is not of infrequent occurrence to find the offspring becoming insane before the parent shows any sign of mental derangement, and the link is often missing which would connect father or mother with insane offspring." We congratulate Nurse Jane Jones and Attendant Avis on having received their pensions, one after 29 years' service and the other after 21 years. There are a few private patients in the asylum and these pay 16s. a week. The average cost per head is 8s. 11d. a week.

### CHESTER COUNTY ASYLUM AT MACCLESFIELD.

Here the numbers increased from 703 to 728 during the year 1896. There were 144 first admissions, 23 readmissions, 56 recoveries, 13 discharged relieved, and 44 deaths. The recoveries, calculated on the admissions, were 35 per cent., and the deaths, calculated on the average number resident, were 6·1 per cent., the latter being exceptionally low. Of the deaths, 21 were caused by cerebral and spinal diseases 10 by consumption, and 1 by enteritis. Of the year's admissions, 52 owed their insanity to various moral causes, 38 to intemperance in drink, and 21 to hereditary influence. Dr. Sheldon discusses at considerable length the real or supposed increase of insanity in these days, and he arrives at the conclusion "that a certain proportion, if not all, of the increase in the number of admissions must be attributed to the diminishing use of workhouses as places of treatment,' and there is no doubt that much of the increase in the number of the registered insane is owing to accumulation rather than to creation; but we are inclined t think that when every possible allowance has been made for the transference of cases from the workhouses, the 4s. capitation grant and the new Lunacy Act, there is a slight increase of insanity among us. Dr. Sheldon also discusses the question of that disease which has made its appearance in our asylums during the last thirty years, to which various names have been given, and which Dr. Sheldon thinks is identical with dysentery. That it has points in common with dysentery there can be no doubt, but we do not think it is identical, and the usual treatment for dysentery seems of little use in the asylum disease. Many years ago the disease was referred to in these columns, and we said it almost seemed as if a new disease had been generated. Since then no fresh light had been shed on it, and it has been attributed to all manner of causes; but it is probable that not one of these is correct, and certainly not one explains the fact that it attacks the insane out of all proportion more frequently than the sane. Thirty of the staff, who had been instructed by Drs. Cooke, Laing, and Bremner, presented themselves for the examination of the Medico-Psychological Association and they all passed. We congratulate Nurses Bayley and Calow on having obtained their pensions. The visitors say they are pleased to report that the asylum continues to be well and ably managed by Dr. Sheldon and the other officers. The asylum farm must be an extremely profitable one for it had a favourable balance of £710; but no doubt farming can be made to pay when nothing is charged for rent, interest on capital, or patients' labour. The average weekly cost per head was 8s. 11⅝d.

### THE HULL BOROUGH ASYLUM.

On January 1st, 1896, this asylum contained 402 patients, and on December 31st the number had risen to 430. There were 126 first admissions, 27 readmissions, 47 recoveries, 22 discharged relieved, and 51 deaths. The recoveries calculated on the admissions stand at 33·33 per cent., and the deaths calculated on the average number resident at 9·18 per cent. There were 18 deaths from general paralysis and 8 from other cerebral or spinal causes, 7 from consumption, and 1 from dysentery. Intemperance in drink accounts for 27 of the admissions, hereditary influence for 34, congenital defect for 11, and previous attacks for 29. When this asylum was opened in 1844 it contained 233 patients, and it now contains 430. Dr. Merson states that the rate of increase shows no sign of falling off, and indeed it cannot be expected to fall off when we think of the ever-increasing population of the Borough of Hull, and of the greater willingness on the part of patients' friends and greater readiness of authorities to send the insane to asylums than was the case fifteen or twenty years ago. The average cost per head per week was a fraction over 11s., but Dr. Merson explains that in this asylum some things are charged to maintenance that are usually charged to building, and with these deductions the rate would not be unduly high.

### CHESTER COUNTY ASYLUM AT UPTON.

Here the numbers increased from 639 to 664. There were 127 first admissions, 42 readmissions, 55 discharged recovered, 5 discharged relieved, and 80 deaths. The recoveries stand at 37·1 per cent. of the admissions, and the deaths at 12·3 on the average number resident. The former is a very fair recovery rate, while the latter is rather above the average of kindred institutions. In 32 instances moral causes contributed to the attacks of insanity in the year's admissions; intemperance in drink was the cause in 19 and hereditary tendency in 28. Of the deaths 43 were owing to cerebral and spinal diseases, 14 to consumption, 3 to pneumonia, and 2 to enteric fever. Dr. Lawrence joins his fellow medical superintendents in commenting on the unfavourable nature of the admissions, and it is certain that many of them were from the first hopeless. Fourteen paralytics, 14 epileptics, and a great many senile cases were certainly rather disheartening raw material. An epidemic of enteric fever broke out which must have taxed the resources of the asylum, as there were two cases of scarlet fever at the same time. There were in all thirty-three cases of typhoid and five deaths; two of these being members of the staff. The outbreak was traced to the pollution of the drinking water. Nursing lectures are given to the staff by Drs. Renton and Ambler, the two assistant medical officers. The farm accounts show a balance of £396; but again we have to notice that no rent is charged, no interest on capital, no sum for patients' labour. The average weekly cost per head is a fraction under 6s. 8d., and while admitting that careful management has much to do with a low rate, we can hardly help thinking that 6s. 8d. is too low.

### THE STAFFORD COUNTY ASYLUM AT STAFFORD.

During the year 1896 the number of patients increased from 876 to 937. There were 370 first admissions, 70 readmissions, 122 recoveries, 139 discharged relieved, and 118 deaths. The recoveries stand at 30·5 per cent. on the admissions, and the deaths at 12·66 per cent. on the average number resident. Of the deaths 44 were due to cerebral diseases, 26 to consumption, 2 to pneumonia, and 3 to enteritis. Table X. shows that domestic trouble, adverse circumstances, religion, &c., caused 74 of the year's admissions, while intemperance in drink was the assigned cause in 108, and hereditary influence in 111. The asylum has been overcrowded during the year, and it is at present very difficult to find vacant accommodation in any county asylum in England. The new Stafford Asylum at Cheddleton is not likely to open its doors for another two years at least, and in the meantime the pressure will continue to increase so much that it was doubtful policy to abandon the idea of building for fifty patients. This would have raised the Stafford Asylum to about 1,000 beds, beyond which, we are convinced, no asylum should be allowed to grow. There is a balance in favour of the farm of £51. Rent is charged, and the prices given of articles supplied to the house. The cost per head per week is 8s. 11½d.

### DERBY BOROUGH ASYLUM.

Here we note a slight decrease in the numbers, there being 311 patients in residence on January 1st, and only 305 on December 31st. There were 59 first admissions, 10 readmissions, 34 recoveries, 7 discharged relieved, and 32 deaths. The recoveries reach the very high percentage of 51·5 on the admissions, and the deaths are about the average at 10·2 per cent. on the average number resident. Of the deaths 17 were caused by cerebral and spinal diseases and 4 by consumption. Sixteen of the year's admissions owed their insanity to moral causes, such as domestic trouble, religion, love, &c., 18 to intemperance in drink, and 17 to hereditary tendency. The slight decrease in the numbers is not due to any falling off in the admissions, but to the high recovery rate and the average death-rate, and it is not likely that Derby will be long exempt from that increase in the number of the registered insane which is the oft-repeated complaint in nearly every county and borough in England. In the meantime Derby is making hay while the sun shines by admitting out-borough patients, and as 100 of these are now in residence the borough must be saving a nice little sum for that rainy day when the asylum is full of its own cases. There is no doubt that the asylum is exceptionally well-managed by Dr. Macphail, and we note that the visitors say they have pleasure in renewing the expression of their entire satisfaction with the manner in which he continues to discharge his responsible and onerous duties. The farm accounts show a balance of £102. Rent of land is charged, but no prices given of articles supplied to the house. The charge to the borough is 9s. 11d. per head per week. Out-borough cases pay 14s., and private patients, of whom there are 16, pay 17s. 6d.

